# Association of Drug Transporter Expression with Mortality and Progression-Free Survival in Stage IV Head and Neck Squamous Cell Carcinoma

**DOI:** 10.1371/journal.pone.0108908

**Published:** 2014-10-02

**Authors:** Rolf Warta, Dirk Theile, Carolin Mogler, Esther Herpel, Niels Grabe, Bernd Lahrmann, Peter K. Plinkert, Christel Herold-Mende, Johanna Weiss, Gerhard Dyckhoff

**Affiliations:** 1 Experimental Neurosurgery Research, Department of Neurosurgery, University of Heidelberg, Heidelberg, Germany; 2 Molecular Cell Biology Group, Department of Otorhinolaryngology, Head and Neck Surgery, University of Heidelberg, Heidelberg, Germany; 3 Department of Clinical Pharmacology and Pharmacoepidemiology, University of Heidelberg, Heidelberg, Germany; 4 Tissue Bank of the National Center for Tumor Diseases (NCT), University of Heidelberg, Heidelberg, Germany; 5 Institute of Pathology, University of Heidelberg, Heidelberg, Germany; 6 Department of Medical Oncology, National Center for Tumor Diseases, University of Heidelberg, Heidelberg, Germany; 7 Hamamatsu Tissue Imaging and Analysis Center, BIOQUANT, University of Heidelberg, Heidelberg, Germany; West German Cancer Center, Germany

## Abstract

Drug transporters such as P-glycoprotein (*ABCB1*) have been associated with chemotherapy resistance and are considered unfavorable prognostic factors for survival of cancer patients. Analyzing mRNA expression levels of a subset of drug transporters by quantitative reverse transcription polymerase chain reaction (qRT-PCR) or protein expression by tissue microarray (TMA) in tumor samples of therapy naïve stage IV head and neck squamous cell carcinoma (HNSCC) (qRT-PCR, n = 40; TMA, n = 61), this in situ study re-examined the significance of transporter expression for progression-free survival (PFS) and overall survival (OS). Data from The Cancer Genome Atlas database was used to externally validate the respective findings (n = 317). In general, HNSCC tended to lower expression of drug transporters compared to normal epithelium. High *ABCB1* mRNA tumor expression was associated with both favorable progression-free survival (PFS, p = 0.0357) and overall survival (OS, p = 0.0535). Similar results were obtained for the mRNA of *ABCC1* (MRP1, multidrug resistance-associated protein 1; PFS, p = 0.0183; OS, p = 0.038). In contrast, protein expression of ATP7b (copper transporter ATP7b), mRNA expression of *ABCG2* (BCRP, breast cancer resistance protein), *ABCC2* (MRP2), and *SLC31A1* (hCTR1, human copper transporter 1) did not correlate with survival. Cluster analysis however revealed that simultaneous high expression of *SLC31A1*, *ABCC2*, and *ABCG2* indicates poor survival of HNSCC patients. In conclusion, this study militates against the intuitive dogma where high expression of drug efflux transporters indicates poor survival, but demonstrates that expression of single drug transporters might indicate even improved survival. Prospectively, combined analysis of the ‘transportome’ should rather be performed as it likely unravels meaningful data on the impact of drug transporters on survival of patients with HNSCC.

## Introduction

Chemotherapy with classical cytostatics such as antimetabolites, platinum drugs, or taxanes remains a cornerstone in the therapy of head and neck squamous cell carcinoma (HNSCC) [1]. Unresponsiveness to antineoplastic agents is frequently due to a phenomenon called multidrug-resistance (MDR) [2]. The classical MDR phenotype is mediated by ATP-binding cassette (ABC)-transporters such as P-glycoprotein (Pgp, *ABCB1*), breast cancer resistance protein (BCRP, *ABCG2*), or several multidrug-resistance-associated proteins (MRPs, *ABCC* family). These membrane-located proteins extrude anticancer agents or their metabolites from cells mediating drug resistance [2]. Paclitaxel, cisplatin, and 5-fluorouracil (5-FU) are standard anti-HNSCC drugs [1], the efficacies of which are limited by several ABC-transporters at least in vitro [3]–[8]. In contrast to experimental studies, clinical data on the role of these proteins is less clear, although some studies for other tumor entities indeed indicated that ABC-transporters negatively influence clinical response or survival of patients suffering from tumors of the lung [9]–[10], the breast [11]–[12], the liver [13], or the kidney [14]. For HNSCC, the significance of ABC-transporters is even more uncertain. First, expression levels have been reported to range from very low [15]–[16] to high expression [17]. Second, impact on chemotherapy response and survival is also inconsistent. MRP1 expression in nasopharyngeal carcinomas was reported to predispose for recurrence and metastasis and to indicate poor 5-year-survival [18]. On the other hand, MRP1 was also documented not to correlate with drug sensitivity or lymph node metastasis [19]. MRP2 and Pgp expression even indicated favorable local tumor control and improved overall survival, respectively [20]. In addition to ABC-transporters such as MRP2, efficacy of cisplatin is also influenced by transporters physiologically involved in copper homeostasis. Human copper transporter 1 (hCTR1/*SLC31A1*) mediates the cellular uptake of copper, cisplatin, and oxaliplatin [21]. The P-type ATPase ATP7b (Wilson disease protein) is also associated with transport of and resistance to cisplatin in vitro, inferior clinical response to cisplatin chemotherapy, and poor survival of HNSCC patients [22]–[23].

We therefore determined expression levels of important drug transporters in tumor specimens of therapy-naïve patients with stage IV HNSCC using quantitative reverse transcription real-time polymerase chain reaction, tissue microarray approach, and external validation controls in order to re-examine contradictory findings by others [20]. The aim of this study was to gain a concluding overview on the significance of all these different drug transporters for progression-free and overall survival times of HNSCC patients.

## Materials and Methods

### Patients

Samples from HNSCC tumor patients and normal mucosa samples from non-cancer patients who underwent tonsillectomy were obtained from the Tissue bank of the National Center for Tumor Diseases (NCT, Heidelberg, Germany) (project no. 374). The study was approved by the institutional ethics committee and written informed consent was obtained from each patient. Clinical data of patients were assessed in an MS Access database (Microsoft, Redmond, USA). Clinical staging and follow-up data were obtained by reviewing the medical records, radiographic images and either by telephone or written correspondence. Patients included did not receive chemo- or radiotherapy prior to surgery. Patients were followed from date of first diagnosis to the end of study, whereas patients who were still alive were censored. Before further use, a vital tumor cell content ≥70% was confirmed on hematoxylin and eosin stained sections by an experienced pathologist of the National Cancer for Tumor Diseases (NCT) Tissuebank.

### Quantification of mRNA expressions by quantitative real time reverse transcription polymerase chain reaction (qRT-PCR)

To exclude possible prognostic confounders and to analyze a rather homogeneous qRT-PCR study sample in the univariate survival analysis all included patients were in clinical stage IVa (Table 1). Drug transporter gene expression was quantified by qRT-PCR. RNA was isolated from tumor specimen using RNeasy-Kit (Qiagen, Hilden, Germany) and cDNA was synthesized with the Transcriptor First Strand cDNA Synthesis Kit (Roche Applied Science, Mannheim, Germany) according to the manufacturers' instructions. Expression of mRNA was quantified by qRT-PCR with a LightCycler 480 (Roche Applied Science, Mannheim, Germany) using the SYBR Green format with the Absolute QPCR SYBR Green Mix. Primer sequences were published previously [24]. The following genes were quantified: *ABCB1*, *ABCC1*, *ABCC2*, *ABCG2*, *SLC31A1*, and *ATP7b*. The most suitable housekeeping gene for normalization was identified using geNorm (version 3.4, Center for Medical Genetics, Ghent, Belgium) [25]. Among the housekeeping genes tested (*β2-microglobulin; glucose-6-phosphate dehydrogenase, G6PDH; glucuronidase β, GU; ribosomal protein L13, RPL13; hypoxanthine-phosphoribosyltransferase 1, HPRT; 60S human acidic ribosomal protein P1*, *HUPO, GU* proved to be the most stable one for this data set. Data were evaluated by calibrator-normalized relative quantification with efficiency correction using LightCycler 480 software as published previously [26]. Results are expressed as the ratio target gene/housekeeping genes divided by the corresponding ratio of the calibrator (equivolumetric mixture of all samples). All samples were amplified in duplicate. Patient characteristics are shown in Table 1.

**Table 1:** Clinical characteristics of qPCR including n = 40 HNSCC patients used for correlation of protein expression with survival.

	Mean ± SD	Range
**Characteristics**		
Age (years)	59±9	46–85
OS (weeks)	186±164	15–886
PFS (weeks)	167±150	6–670
	**Frequency**	**Percent**
**Gender**		
M	35	87.5
F	5	12.5
**Localisation**		
Oropharynx	21	52.5
Hypopharynx	11	27.5
Larynx	3	7.5
Oral Cavity	5	12.5
**Therapy**		
Radiation	5	12.5
Chemotherapy	11	27.5
Radio/Chemotherapy	24	60.0
Surgery	40	100.0
**Clinical stage**		
I–II	0	0.0
III–IV	40	100.0

### Quantification of ATP7b expression by tissue microarray (TMA)

Formalin-fixed, paraffin-embedded tissue samples from 87 patients were used for TMA design (Table 2). Representative tumor regions were identified by an experienced pathologist on H&E-stained tissue sections. From all selected regions, tissue cylinders with a diameter of 0.6 mm were obtained and arrayed into a recipient block as described earlier [27]. The recipient block was subsequently cut into 5 µm sections on precleaned microscope slides (Superfrost Plus, Thermo Scientific, Braunschweig, Germany).

**Table 2:** Clinical characteristics of TMA including n = 61 HNSCC patients used for correlation of protein expression with survival.

	Mean ± SD	Range
**Characteristics**		
Age (years)	59±10	35–87
OS (weeks)	191±140	5–544
PFS (weeks)	162±143	5–520
	**Frequency**	**Percent**
**Gender**		
M	51	85.0
F	9	15.0
**Localisation**		
Oropharynx	29	47.5
Hypopharynx	15	24.6
Larynx	14	23.0
Oral Cavity	3	4.9
**Therapy**		
Radiation	12	19.7
Chemotherapy	24	39.3
Radio/Chemotherapy	25	41.0
Surgery	61	100.0
**Clinical stage**		
I–II	8	13.1
III–IV	53	86.9

Prior to TMA staining specificity of primary ATP7b antibody was ensured using an isotype control (PP501P, Acris, Hiddenhausen, Germany). Proceeding staining deparaffinization was carried out by immersing slides in 100% xylol (3×3 min), followed by 90%, 80%, 70% and 50% ethanol (2×3 min each). Finally, slides were washed in distilled water (2×3 min). Antigen retrieval was performed in an autoclave at 1 bar, 125°C for 20 min using antigen retrieval buffer (DAKO, Hamburg, Germany) at pH 6.1. Incubation with primary and secondary antibodies as well as detection with Vectastain ELITE ABC Kit (Vector Laboratories, Burlingame, USA) was carried out as described [28].

Antigen expression was pre-tested in a set of HNSCC tissues to establish suitable antigen evaluation categories based on antigen expression variability. Grading scores with uniform distribution of antigen expression levels among individual grading categories were chosen for the final TMA evaluation. Slides were scanned at a 20× magnification by the Nanozoomer Digital Pathology (NDP) System (Hamamatsu Photonics, Hamamatsu, Japan) by the BIOQUANT TIGA Center of the University Heidelberg. Scanned TMAs were afterwards evaluated with the help of the NDP Viewer software (Hamamatsu Photonics, Hamamatsu, Japan). Each tumor biopsy was scored semiquantitatively on the basis of a well-established immunoreactivity scoring system (IRS) [29]. Each investigator (DT, JW) ranked a value for the expression intensity from 1 (no staining) to 4 (very strong expression) and a value describing the extent of tumor staining (1, no expression; 2, 0–24%; 3, 25–49%; 4, 50–74%, 5, 75–100%). These values were multiplied. The final score of each tumor was then calculated as the mean of these two independent evaluations. Consequently, lowest score was 1, highest score was 20.

### Data base query

The Cancer Genome Atlas (TCGA; URL: https://tcga-data.nci.nih.gov/tcga/) validation data were retrieved as level 3 normalized RNAseq gene expression files. Age, sex, clinical staging, survival times, vital status, drug and radiation treatment were annotated clinical data provided by TCGA. Only patients of clinical stage II-IVa were selected for survival analyses. Patient characteristics are shown in Table 3.

**Table 3:** Clinical characteristics of TCGA dataset including n = 317 HNSCC patients used for correlation of RNAseq mRNA expression with survival.

	Mean ± SD	Range
**Characteristics**		
Age (years)	61±12	19–90
OS (weeks)	113±124	1–917
PFS (weeks)	98±93	6–561
	**Frequency**	**Percent**
**Gender**		
M	230	72.6
F	87	27.4
**Localisation**		
Oropharynx	42	13.2
Hypopharynx	3	0.9
Larynx	77	24.3
Oral Cavity	195	61.5
**Therapy**		
Radiation	39	12.3
Chemotherapy	0	0.0
Radio/Chemotherapy	72	22.7
Surgery	317	100.0
**Clinical stage**		
II	70	22.1
III	81.00	25.5
IV	166.00	52.4

### Statistical analysis

Differences in drug transporter expression levels between tumor and control samples were analyzed using two-sided t-tests. RNAseq data were analyzed using Mann-Whitney U test. Probability values of p<0.05 were considered significant. Survival associations were calculated using a log rank test and displayed as Kaplan-Meier plots. Calculations were performed using Prism 5 statistics software (GraphPad Software, La Jolla, USA). Heatmaps were drawn using the SUMO statistic software (http://angiogenesis.dkfz.de/oncoexpress/software/sumo/).

## Results

### mRNA expression of drug transporters in HNSCC

**Figure 1:**
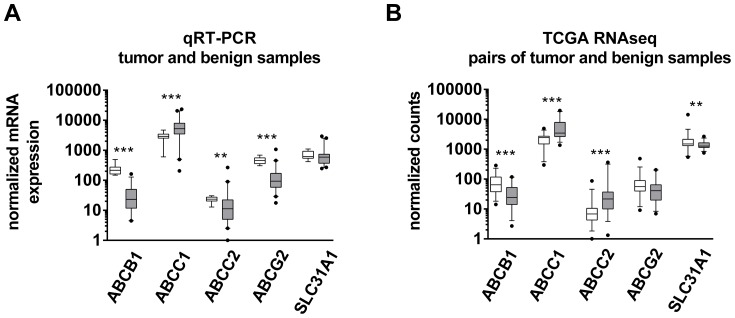
mRNA expression of drug transporters in HNSCC. (A) Determination of mRNA expression levels of drug transporters by qRT-PCR in a study sample of therapy naïve stage IVa HNSCC tumors (n = 40, grey boxes) and normal control samples (n = 14, white boxes) (relative mRNA expression normalized to the lowest value). (B) External validation by HNSCC RNAseq mRNA expression data derived from the ‘The Cancer Genome Atlas’ (TCGA) consortium. Comparison of normalized counts of paired tumor (n = 37, grey boxes) and adjacent noncancerous normal tissue (n = 37, white boxes). Whisker indicates 5–95 percentile; Mann-Whitney U test; **, p<0.01; ***, p<0.001.

mRNA expression levels of a subset of drug transporters (*ABCB1*, *ABCC1*, *ABCC2*, *ABCG2*, *SLC31A1*) were determined by qRT-PCR in a study sample of therapy naïve stage IVa HNSCC tumors (n = 40) and normal control samples (n = 14) (Fig. 1A). In general, *ABCB1* and *ABCC2* were very low expressed in HNSCC, whereas *ABCC1* was highly expressed. *ABCG2* and *SLC31A1* exhibited intermediate mRNA expression levels. Comparing tumor and normal tissues, three of the five analyzed genes showed a significant lower expression in tumors than in healthy control tissues (*ABCB1*, p<0.0001; *ABCC2*, p<0.005; *ABCG2*, p<0.0001), whereas *ABCC1* expression was significantly up-regulated in the carcinomas (p<0.0001). These differences resulted in a 9.4- , 2.1- and 4.9-fold lower median expression of *ABCB1*, *ABCC2* and *ABCG2* in tumor samples compared to control samples, respectively. *ABCC1* exhibited a 1.8-fold higher median expression in tumor samples. *SLC31A1* expression did not differ between HNSCC and control samples.

To validate our findings in an independent dataset, we extracted RNAseq mRNA expression data from the HNSCC database derived from the TCGA consortium (Fig. 1B). *ABCB1* (p<0.0001) and *ABCG2* (not significantly) also showed lower expression in tumors, whereas *ABCC1* was also significantly (p<0.0001) higher expressed in tumors. In contrast to our study sample, *SLC31A1* expression was significantly down-regulated in the TCGA dataset of HNSCC (p = 0.0036) and *ABCC2* exhibited significantly higher expression in tumors (p = 0.0002), whereas it was significantly down-regulated in our samples.

### Impact of mRNA expression of drug transporter genes on patient survival

To analyze association of drug transporter gene expression with survival of HNSCC patients, patients were assigned into two groups differing by either higher or lower gene expression than the median of all samples. Based on this grouping the genes showed a divergent correlation with survival in the univariate log rank survival analysis.

**Figure 2:**
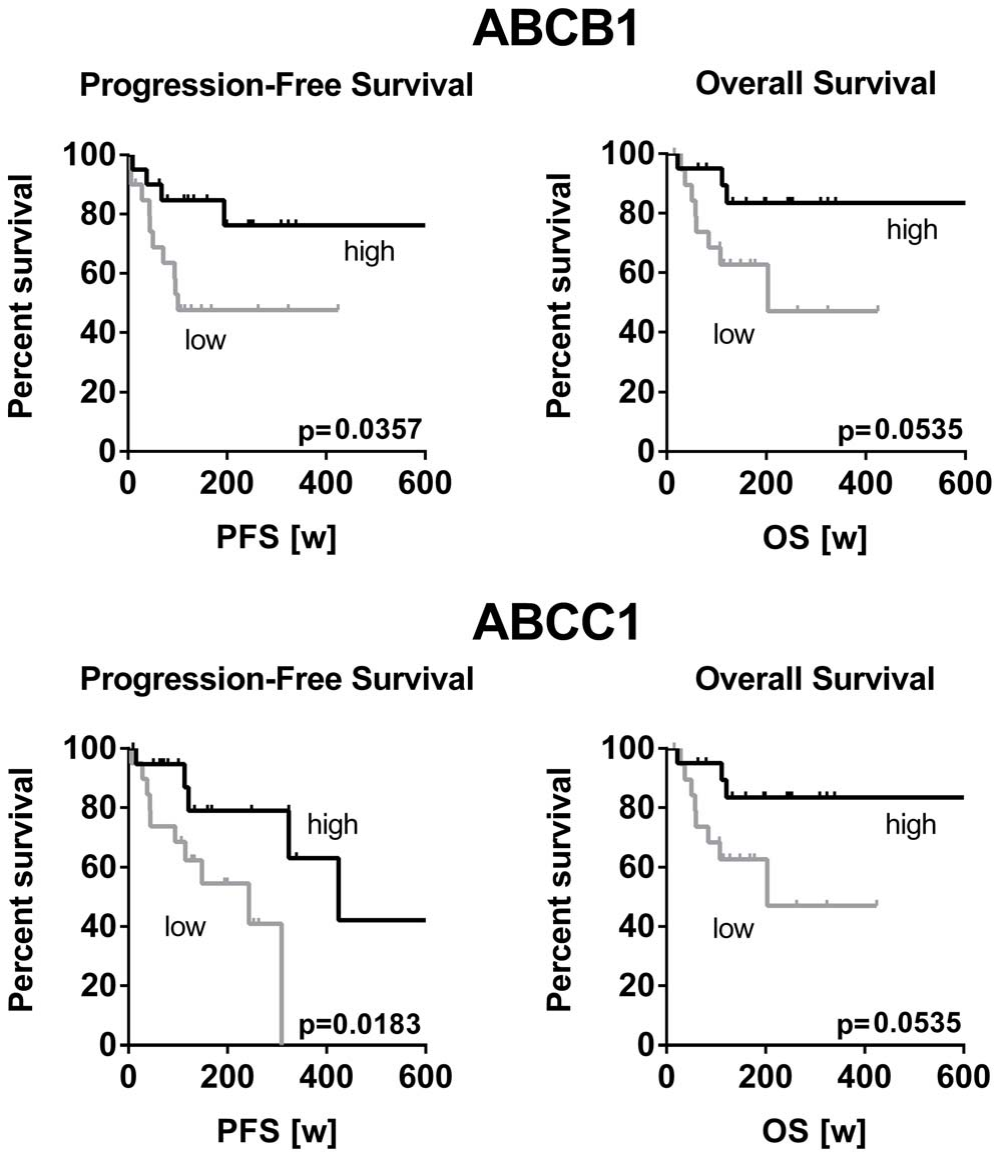
Correlation of low mRNA expression and shortened survival of HNSCC patients. Classification of HNSCC patients according to the gene expression level in the high (n = 20) or low (n = 20) expression group. Survival analysis by Kaplan-Meier curves and log rank test revealed a significant correlation of lower expression and shortened progression-free and overall survival.

**Figure 3:**
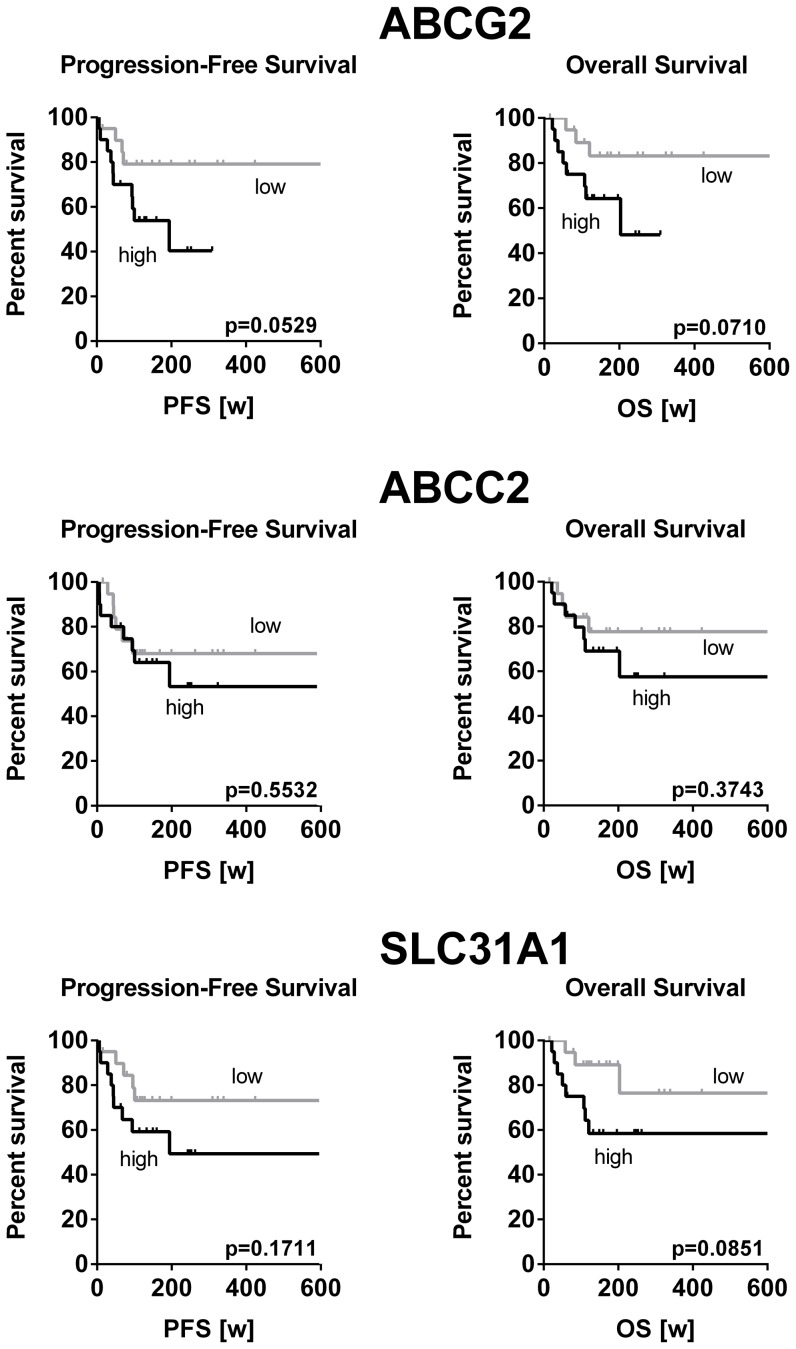
Correlation of high mRNA expression and shortened survival of HNSCC patients. Classification of HNSCC patients according to the expression level of the respective gene in high (n = 20) or low (n = 20) expression group. Survival analysis by Kaplan-Meier curves and log rank test revealed that patients with high gene expression tended to accompany a shorter progression-free and overall survival.

High *ABCB1* as well as high *ABCC1* mRNA gene expression was significantly associated with longer progression free survival (p = 0.0357 and p = 0.0183, respectively) and overall survival (p = 0.0535 and p = 0.038, respectively) (Fig. 2). In contrast, high expression levels of *ABCG2*, *ABCC2* and *SLC31A1* tended to be associated with a shorter survival time of HNSCC patients (Fig. 3). However, these associations did not reach statistical significance.

**Figure 4:**
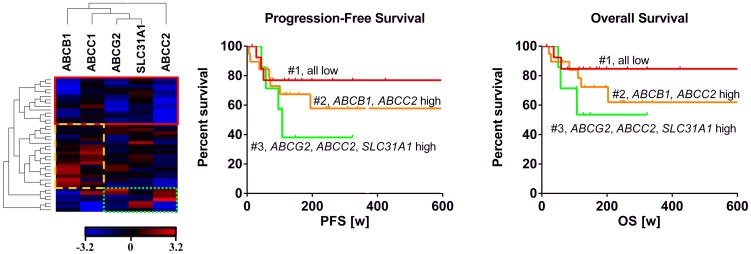
Hierarchical clustering of mRNA expression and survival of HNSCC patients. (A) Heatmap of the median normalized log2 transformed mRNA expressions hierarchically clustered with Euclidean distance matrix and complete linkage. Three distinct groups were uncovered: First group (n = 14) low expression of all drug transporters evaluated (#1, red framed), second group (n = 19) high *ABCB1* and high *ABCC1* expression (#2, yellow framed) and a third group (n = 7) showing low *ABCB1*/*ABCC1* expression but high levels of either *ABCG2*, *SLC31A1*, or *ABCC2* (#3, green framed). (B) Survival analysis by Kaplan-Meier curves and log rank test revealed that patients of group #3 (n = 7) tended to survive shorter than those of group #2 (n = 19). Best survival was seen for patients of group #1 (n = 14) who show a lower expression of drug transporters.

Because there was a substantial difference in expression levels, potential mutual exclusive up- or down-regulation of genes was analyzed through hierarchical clustering the median normalized log2 transformed mRNA expression values. The respective heatmap uncovered three distinct groups (Fig. 4): First group (n = 14) constantly exhibiting low expression of all drug transporters evaluated (#1, red framed), second group (n = 19) with both high *ABCB1* and high *ABCC1* expression (#2, yellow framed) and a third group showing low *ABCB1*/*ABCC1* expression but high levels of either *ABCG2*, *SLC31A1*, or *ABCC2* (#3, green framed). Best survival was observed for the first group with constantly low expression of all drug transporters evaluated. In contrast, lowest survival was observed for the third group of patients with tumors exhibiting high expressions of *ABCG2*, *SLC31A1*, and *ABCC2*. The second group (high *ABCB1* and high *ABCC1*) exhibited intermediate survival times.

### Validation of survival in the independent TCGA dataset

**Figure 5:**
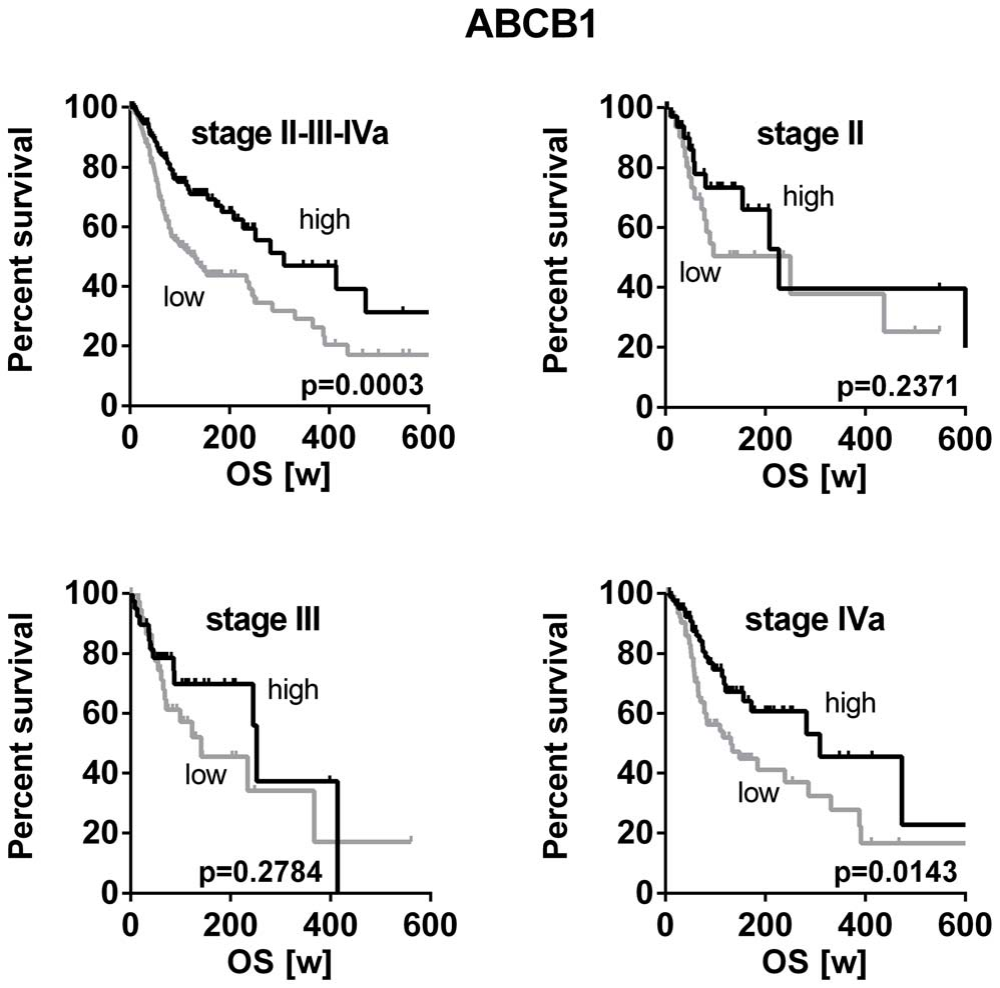
Correlation of RNAseq mRNA expression and overall survival of HNSCC patients in the TCGA dataset. Classification of stage II-III-IVa TCGA HNSCC patients according to the ABCB1 expression level in high or low. Survival analysis by Kaplan-Meier curves and log rank test of all stages together (upper left; high, n = 159; low, n = 158) or either stage II (upper right; high, n = 34; low, n = 36), stage III (lower left; high, n = 40; low, n = 41) and stage IVa (lower right; high, n = 83; low, n = 83) revealed consistently shorter survival times for patients with high ABCB1 expression, which reached significance in the whole group and the subgroup of stage IVa tumors.

To challenge our findings, the publicly available TCGA HNSCC dataset containing high patient numbers of all clinical stages was again used for validation in anindependent dataset. Patients with HNSCC of stage II (n = 70), stage III (n = 81) or stage IVa (n = 166) were again grouped according to our previous approach into lower or higher than the median of all samples, respectively. Consistent with our initial finding, high *ABCB1* expression significantly correlated with a better overall survival (p = 0.0003) (Fig. 5). Stratification according to clinical stage indicates significant correlation with survival within the group of stage IVa patients (p = 0.0143). In stage II and III the same trend was obvious, but it did not reach statistical significance (Fig. 5).

### ATP7b protein expression evaluated by tissue microarray

**Figure 6:**
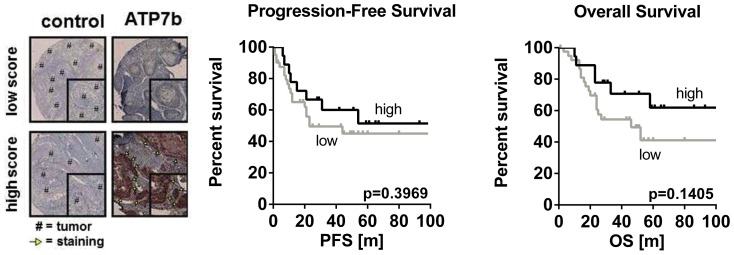
Immunohistochemical staining of ATP7b protein expression in HNSCC. Immunohistochemical staining of ATP7b protein expression in HNSCC patients (n = 61) revealed a high range of immunoreactive scores (left). Grouping of patients according to their immunoreactive scores (cutoff  = 15) into low (n = 41) or high (n = 18) showed a trend towards a longer progression-free and overall survival in patients with a higher ATP7b expression (right).

There was a high variation of ATP7b protein expression among the 61 HNSCC samples evaluated with immunoreactive scores between 1 to 19 (median 14). Grouping of these patients according to their immunoreactive score (cutoff 15) and subsequent survival analysis showed that ATP7b expression levels did not correlate with progression-free survival (p = 0.3969) or overall survival (p = 0.1405), respectively (Fig. 6). However, concordant to *ABCB1* and *ABCC1* patients with high ATP7B protein expression tended to survive longer.

## Discussion

Identification of tissue biomarkers in biopsy specimens of HNSCC may not only select patients that may benefit from more aggressive treatment modalities but may also indicate prognosis. To date, robust clinical, molecular, or radiographic markers in HNSCC are still rare [30]. Therefore, we intended to investigate the expression of drug transporters in these tumors, because their clinical relevance is unclear so far. Besides their role as mediators of cytostatic drug resistance, ABC-transporters have also been proposed as markers of malignancy in HNSCC. In parotid mucoepidermoid carcinoma advanced grades exhibited higher Pgp expression levels than tumors of lower grades [31]. Moreover, Pgp expression has been reported to generally increase during the course of the disease [32]–[33]. Consequently, our samples of higher stage HNSCC were expected to also show higher expression levels of drug transporters than non-tumor controls. However, the opposite was demonstrated. *ABCB1*, *ABCC2*, and *ABCG2* were significantly lower expressed in tumors than in normal epithelium of tonsils. The same trend was observed for *SLC31A1* but without reaching statistical significance. *ABCC1* was the only drug transporter evaluated that was overexpressed in tumors (Fig. 1A). To validate these unexpected findings and to rule out that our observations were biased by unrelated factors, the TCGA dataset was analyzed for drug transporter expression in HNSCC and adjacent non-tumor control tissue. Because here 37 pairs of tumors and their normal counterparts of the very same patient were compared, the results are considered independent of confounders such as gender, age, or smoking status. Except *ABCC2*, the TCGA dataset generally confirmed our results by demonstrating that drug transporters such as *ABCB1* (Pgp) are highly significantly (P<0.0001) down-regulated in HNSCC (Fig. 1B). In contrast to the intuitive dogma, such low expression seems to be associated with a malignant phenotype and advanced tumor disease. This assumption was supported by the subsequent survival analysis. HNSCC patients with low *ABCB1* expression had significantly shorter progression-free survival times and tended to die earlier (overall survival, p = 0.0535) than patients with *ABCB1* expression higher than the median (Fig. 2). This finding was again confirmed using the TCGA dataset by demonstrating that low expression of *ABCB1* correlates with poor overall survival in HNSCC stage II - IVa while high expression of *ABCB1* rather indicates favorable survival. Stratification for tumor stages confirmed this trend and again showed that in advanced tumors (stage IVa), poor survival in association with low *ABCB1* expression (Fig. 5). Mechanistically, it is hard to understand why high expression of *ABCB1* and *ABCC1* was related to improved survival while low expressions indicated poor survival. Recently, experimental studies suggested that overexpression of ABC-transporters leads to enhanced efflux of glutathione and diminished cellular glutathione content. When intracellular glutathione levels are low, platinum drug species are rarely complexed and thus remain pharmacologically active [34]. In consequence, increased expression of glutathione export transporters can indeed promote efficacy of platinum drugs in HNSCC and lead to a clinical benefit. This observation has been confirmed clinically in HNSCC [20]. Second, cancer is frequently accompanied by inflammatory processes in the microenvironment of the tumor, especially with advancing disease [35]–[36]. Secreted inflammation-associated cytokines (e.g. tumor necrosis factors, interleukines, etc) are in turn very well known to subsequently downregulate drug transporters [37]. In consequence, the observed downregulation of drug transporters in HNSCC tissue might simply be an indicator of enhanced inflammation which is typically observed in advanced stages of cancer and accompanied by poor survival [38].

In contrast to *ABCB1* and *ABCC1*, high expression of *ABCG2*, *ABCC2*, and *SLC31A1* tended to indicate poor progression-free survival and overall survival, but none of these associations reached statistical significance (Fig. 3). Due to these contradicting results, a cluster analysis was subsequently performed in order to detect dependencies or expression patterns that concertedly determine survival. Three distinct groups were identified. Best survival was observed when all drug transporters showed a reduced expression, whereas both progression-free and overall survival was shortened with coordinated high expression of *SLC31A1, ABCG2*, and *ABCC2* (Fig. 4). The latter two genes are known cancer-stem cell markers in HNSCC [39]–[40]. In consequence, it is comprehensible that patients with high expression of *ABCG2* and *ABCC2* (and thus potentially high cancer-stem cell burden) exhibit poor survival. Together, the results of the cluster analysis finally suggest that the course of the disease or survival cannot be estimated by a single drug transporter gene, but rather by the whole ‘transportome’ or at least the combination of certain drug transporters (e.g. cancer-stem cell marking drug transporters).

ATP7b expression was evaluated at the protein level using tissue microarray methodology (Fig. 6A). A high variation of the immunoreactive score was observed among the 61 HNSCC samples evaluated. However, survival analysis revealed that ATP7b expression does not correlate with survival times (Fig. 6B) contradicting earlier findings by others [23].

In conclusion, this study contradicts the intuitive dogma whereupon high expression of ABC-transporters such as Pgp is unfavorable for survival of HNSCC patients. In contrast, overexpression of distinct drug transporters might even indicate improved survival. Cluster analysis evaluating a subset of drug transporters including cancer-stem cell markers such as *ABCG2* is suggested for further studies on the significance of drug transporters for HNSCC disease.
